# Commonly Used Outcome Measurement Tools in Pediatric Physical Therapy Telerehabilitation in the Philippines: A Quantitative Cross-Sectional Descriptive Study

**DOI:** 10.5195/ijt.2021.6427

**Published:** 2021-12-16

**Authors:** Arlene Chiong Maya, Therese Daniela Manaloto, Christian Rey Rimando, Maria Eliza Dela Cruz, Daniel Stephen Banting, Alliana Cielo Equipaje, Noel Antonio Ipo, Jana Mae Mosi Ramos, Marc Jefferson Rillas, Jaycelle Anne Tajan

**Affiliations:** Department of Physical Therapy, College of Rehabilitation Sciences, University of Santo Tomas, Manila, Philippines

**Keywords:** Outcome measurement tools, Pediatric physical therapist, Telerehabilitation

## Abstract

With the COVID-19 pandemic, the adoption of telerehabilitation has rapidly increased to improve access and minimize cross-infection risk to patients. Nevertheless, Filipino pediatric physical therapists (PTs) must ensure that they conduct evidence-based procedures for specific tests and measures to determine patient outcomes. This investigation reported the most common pediatric outcome measurement tools (OMTs) used in telerehabilitation by Filipino pediatric PTs treating 0 to 21-year-olds in the Philippines. Validation and pilot testing of an adapted questionnaire on OMT usage was undertaken before dissemination via email and social media. Pediatric PTs reported that the commonly used OMTs in telerehabilitation are Gross Motor Function Measure (GMFM) (100%)—including both versions of GMFM-88 and GMFM-66 followed by Pediatric Balance Scale (PBS) (30%). These findings support the use of feasible OMTs in pediatric telerehabilitation due to their applicability in the online setting.

Pediatric physical therapists (PTs) work with children and their families to provide interventions promoting children's ability to function independently and actively participate in their home, school, and community environments (American Physical Therapy Association [APTA] Pediatrics, 2020). APTA Pediatrics (2020) further states that pediatric PTs aim to improve the motor skills of children with delayed motor development, strength, endurance, balance, and coordination. According to World Physiotherapy (2019), the latest statistics (as of June 30, 2019) reveal that 173,294 physical therapists work in the Asia Western Pacific region, including the Philippines, with 20% specializing in pediatrics. On May 14, 2017, the Philippine Physical Therapy Association (PPTA) launched its own pediatrics special interest group, the PPTA Pediatrics Special Interest Group (PPTA Pedia SIG). It held a seminar on pediatric evaluation tools (PPTA Pedia SIG, 2017). PPTA Pedia SIG follows PPTA's stated summary of the role of a PT, which includes examining individuals or groups through history taking, screening, using specific tests and measures, and re-examining patients or clients as necessary to determine outcomes (Philippine Physical Therapy Association [PPTA], 2020a).

Outcome measurement tools (OMTs) are standardized instruments evaluated for their psychometric properties and used to measure the change in a patient's health status through quantitative assessment of function (Jette et al., 2009). Pediatric PTs utilize OMTs to quantify children's motor skills and functional development. The OMTs serve as a basis to establish a course of treatment necessary for maximal function (Fay et al., 2018). To determine the most appropriate outcome measure, Coster (2013) reported that it is essential to consider dimensions, format, reliability, responsiveness, and feasibility. Common OMTs, according to the American Physical Therapy Association (APTA), include Children's Assessment of Participation and Enjoyment (CAPE) and Preferences for Activities in Children (PAC), CP Functional Classification Systems, and Gross Motor Function Measure (GMFM) (Therapy BC, 2020). The utilization of these instruments in the clinical setting has been effective as these standardized outcome measures provide a common language among physical therapists (APTA, 2020).

During the current COVID-19 pandemic, the adoption of telerehabilitation has rapidly increased to improve access and minimize cross-infection risk to patients (Hwang & Elkins, 2020). Telerehabilitation in the Philippine context refers to remotely conducting evaluation, consultation, therapy, and monitoring using electronic means. This practice delivers rehabilitative care for patients in different locations, including home, community, nearby health facility, and workplace settings (Leochico et al., 2020). Eguia and Capio (2021) reported that video consultations became an essential service delivery strategy for pediatric therapists in response to the COVID-19 lockdown. Zoom has been the most commonly used digital platform for telerehabilitation (72%), followed by Facebook Messenger (28%) and Viber (16%). Reported benefits of telerehabilitation include empowering parents and enhancing their understanding of their children's needs (Eguia & Capio, 2021). Furthermore, PPTA has included transitioning to telerehabilitation in its interim recommendations on PT services during the COVID-19 pandemic (PPTA, 2020b).

There are currently no established, validated, or reliable pediatric OMTs for telerehabilitation. However, some PTs may use validated OMTs in the in-person setting for telerehabilitation (Domingo, 2020). A study that established a foundation for efficient and effective pediatric telerehabilitation visits in response to the barriers caused by the pandemic also recommended this strategy (Rabatin et al., 2020).

The recommended OMTs for pediatric telerehabilitation include: the Physician Rating Scale (PRS), Edinburgh Visual Gait Analysis, Modified Wisconsin Gait Scale, Modified Physician Global Assessment Scale (PGA), Penn Spasm Frequency Scale, and the Spinal Cord Injury Spasticity Tool (SCI-SET). Moreover, assessments in adults receiving assistance from caregivers or self-administering OMTs in the telerehabilitation setting were generally feasible, supported by good validity and reliability, excluding lumbar spine posture and some orthopedic tests (Rabatin et al., 2020).

In Metro Manila, Zotomayor (2018) investigated the initial use of OMTs by analyzing 96 initial evaluation charts. Three standard OMTs with good psychometric properties were recommended, including GMFM, Pediatric Evaluation of Disability and Inventory (PEDI), and Activities Scale for Kids (ASK). However, the study revealed that only 18.75% of the 96 initial evaluation charts reported using GMFM. Zotomayor (2018) further noted that there is currently limited research on OMTs in the telerehabilitation setting, particularly in pediatric physical therapy. Thus, the purpose of this study was to determine the most commonly used pediatric OMTs in telerehabilitation by Filipino pediatric PTs in the Philippines.

## MATERIALS AND METHODS

### RESEARCH DESIGN

This research is a cross-sectional descriptive quantitative study registered under the Philippine Health Research Registry (PHRR210318-003267). The study has two phases: Phase I: Validation and Pilot Testing of the Adapted Questionnaire and Phase II: Questionnaire Implementation.

### ETHICAL CONSIDERATIONS

The Ethics Committee of the University of Santo Tomas-College of Rehabilitation Sciences (CRS-ERC) reviewed and approved this study with the approved protocol number SI-2020-034. The study abided by the Declaration of Helsinki, National Ethical Guidelines for Health and Health-Related Research of Philippine Health Research Ethics Board, and Data Privacy Act of 2012 (RA 10173). The researchers fully informed all recruited participants of the objectives, duration, procedures, benefits, risks, and record-keeping of the study before consenting to participate.

### PARTICIPANTS

#### PHASE I: VALIDATION AND PILOT TESTING OF THE ADAPTED QUESTIONNAIRE

Phase I included a panel of three experts (consisting of one pediatric telerehabilitation practitioner with at least one year of experience and two methodologists or experts in questionnaire validation with at least five years of experience) to validate the content of the adapted questionnaire using the content validity index (CVI). The number of experts (n = 3) was deemed adequate for content validation since three to 10 experts is considered the accepted range for raters (Rodrigues et al., 2017). After validation, the researchers conducted pilot testing to determine the survey's duration and obtain feedback on the survey's online format. Experts recommended that the sample size for the pilot test should be 10% of the actual study's projected participants (Connelly, 2008). Hence, the pilot testing included six practicing Filipino pediatric telerehabilitation PTs treating 0 to 21-year-old patients.

#### PHASE II: QUESTIONNAIRE IMPLEMENTATION

The study recruited 45 eligible Filipino pediatric telerehabilitation PTs treating 0 to 21-year-old patients for phase II. Demographic characteristics of the participants included male or female pediatric PTs aged from 18 years old and above with years of practice as a telerehabilitation practitioner ranging from less than one to three, who may or may not have been using OMTs. Purposive sampling to remove sampling bias was used for pilot testing and in Phase II by inviting all PPTA Pedia SIG members who met the inclusion criteria. The study also employed snowball sampling and convenience sampling by sending invitations to pediatric rehabilitation centers and clinics throughout the country through email or social media. The pilot testing and Phase II excluded Filipino pediatric PTs practicing abroad to focus on the current use of OMTs by pediatric PTs based in the Philippines. Nevertheless, the study was not limited to recruiting pediatric PTs treating particular pediatric conditions.

### SAMPLE SIZE

The researchers computed the sample size for Phase II utilizing the sample size calculator from the Creative Research Systems Software, software established in 1982 with a reputation for developing leading-edge survey software for various professionals, including researchers using questionnaires (Creative Research Systems, 2012). The group set the confidence level at 95% and 8% for the margin of error. The population of PPTA Pedia SIG stood at 100 members based on its database as of June 5, 2021. Therefore, the researchers recruited at least 60 participants based on the calculated result.

### SETTING

The researchers conducted the study via online platforms. Facebook and Gmail were used to send invitations for participant recruitment. The research development primarily occurred in the National Capital Region (NCR), but respondents participated from different locations within the Philippines.

### INSTRUMENT

A tool formulated by Jette et al. (2009) was adapted. The author granted permission to use the questionnaire in March 2021. Multiple published studies using the same methodology have similarly adapted the tool (Hasani et al., 2020; Valier et al., 2014). These studies determined the use of standardized OMTs and perceptions on the benefits of and barriers to their use.

In terms of adaptation, the only questions taken from the original questionnaire were those considered relevant to the study's objective (Appendix A). The study disregarded questions on perceived barriers and benefits of OMT use and the methodology of OMTs used since they were not within the scope of the study.

For OMT users, 18 items were taken from the original questionnaire and split into five sections. The items used include Numbers 1-2 from Section 1: Demographics; 47-49 from Section 3: Policies and Procedures for OMT Use in Practice Setting; 63-67 from Section 4: List of OMTs Used in Telerehab; and 53-60 from Section 5: Criteria Used for OMT Selection. Item numbers 53-60 were synthesized into one question with eight statements. The researchers added Section 2: Usage or Non-Usage of OMTs to direct participants to the survey content appropriate for them. Furthermore, the study added fields for the email address, age, and years of experience as a pediatric PT using telerehabilitation in the demographics section. Hence, the adapted questionnaire for OMT users consisted of 15 items. The estimated time to complete the survey was approximately 10 to 15 minutes.

For non-OMT users, the adapted questionnaire included all 21 reasons for the non-usage of OMTs from the original questionnaire. The survey for non-OMT users was comprised of eight questions with three sections: Demographics, Usage or Non-Usage of OMTs, and Reasons for Non-Usage of OMTs. The estimated time for non-OMT users to complete the survey was only 5 to 10 minutes.

For both phases of the study, the researchers utilized Google Form, where they applied the “required” option to all questions to avoid missing data. Each form contained the participant information sheet (PIS) and informed consent form (ICF), along with the items mentioned above. Completing the survey in the context of telerehabilitation was emphasized in the survey's instructions. In Phase I, the respondents were required to input their start and end times for time tracking purposes. The researchers included a comments section at the end of the pilot test survey to obtain feedback on the Google Form format.

### PROCEDURES

**Figure 1:**
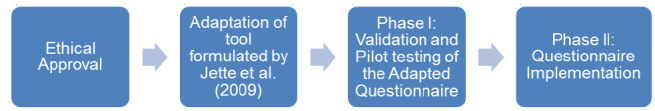
Procedures of the Study

Figure 1 illustrates the procedures of this study. The study invited a panel of three experts to validate the adaptation of the questionnaire formulated by Jette et al. (2009). An item validity form via Google Form asked each expert to rate each item of the adapted questionnaire, using a 4-point Likert scale, as highly relevant (4), quite relevant (3), somewhat relevant (2), or not relevant (1). The Item-Content Validity Index (I-CVI), Scale-Content Validity Index using the Universal Agreement method (S-CVI/UA), and Scale-Content Validity Index using the Average method (S-CVI/Ave) were computed to determine the validity of each item and the questionnaire's overall validity (Rodrigues et al., 2017).

Researchers coordinated with PPTA Central and PPTA Pedia SIG to allow the dissemination of information on the study. Additionally, they created a directory of pediatric centers and clinics in the Philippines offering telerehabilitation to recruit more respondents. Publication materials and invitation letters, including a sign-up link via Google Form, were sent to PPTA Pedia SIG, pediatric centers, and clinics where interested participants input their contact information.

The pilot testing included the first six participants who registered. They received a link containing the PIS, ICF, and the survey via email or social media. The researchers sent out follow-up notifications in cases where a participant could not respond within five days to remove non-response bias. They sent the notifications on the Saturday of the second to fourth weeks of June 2021. The study excluded the participants who did not respond after this period.

Phase II included the remaining registered participants, and the study employed the same procedures. After the period of data gathering, the researchers restricted the accessibility of the online survey.

The researchers stored the online survey results in a password-protected Google Drive and utilized Google's two-step verification feature to minimize the risk of loss of confidentiality. The Google Drive with the gathered information was only accessible to an indicated researcher and the study's statistician.

### STATISTICAL METHODS

For Phase I, the study determined the content validity of the adapted questionnaire measured using CVI. The statistician computed for the I-CVI to determine the content validity of individual items and S-CVI/UA and S-CVI/Ave to determine the content validity of the overall scale. Before the I-CVI computation, the statistician translated the relevance rating as 1 (relevance scale of 3 or 4) or 0 (relevance scale of 1 or 2). The recommended acceptable cut-off score of I-CVI with three experts should be 1 (Yusoff, 2019). For the S-CVI, researchers suggest that a scale have an S-CVI/UA ≥ 0.8 and S-CVI/Ave ≥ 0.9 to have excellent content validity (Polit & Beck, 2006). Table 1 illustrates the formula of the I-CVI, S-CVI/UA, and S-CVI/Ave. Moreover, the pilot testing for Phase I determined the survey's average duration among the six participants. The researchers sought feedback on the online questionnaire format and no revisions were necessary.

**Table 1:** The Formula of I-CVI, S-CVI/UA, and S-CVI/Ave

The CVI indices	Formula
I-CVI	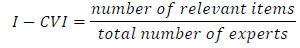
S-CVI/UA	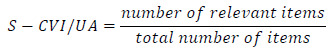
S-CVI/Ave	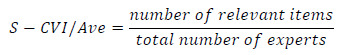

*Note.* I-CVI = Item-Content Validity Index; S-CVI/UA = Scale-Content Validity Index using the Universal Agreement method; S- CVI/Ave = Scale-Content Validity Index using the Average method

For Phase II, the study utilized the Microsoft Excel version 16.54 and SPSS Software version 22 for data analysis (IBM SPSS Statistics, 2020). The researchers interpreted the answers gathered from the demographic and close-ended questions using frequency and percentage. Similarly, the statistician tallied the responses and determined response frequencies to identify the common OMTs used in pediatric telerehabilitation. The study reported the data from Phase II using graphical, tabular, and textual methods.

## RESULTS

### PHASE I: VALIDATION AND PILOT TESTING OF THE ADAPTED QUESTIONNAIRE

The original version of the questionnaire underwent two phases of amendment: (1) original, adapted version and (2) final validated version (Appendix A). The analysis of the CVI ratings from the panel of three experts rendered the results summarized in Table 2. Appendix B shows the per item CVI analysis. The initial version of the adapted questionnaire consisted of 15 items for the OMT users and eight items for the non-OMT users during telerehabilitation. Since one item for the OMT and non-OMT users is comprised of multiple response questions, the statements were rated instead of the actual question. Hence, after going through the CVI analysis, the final version of the questionnaire retained the same number of items. The researchers eliminated statements two and seven from the multiple response questions for OMT and non-OMT users, respectively, as they did not meet the cut-off criteria set in the study (Appendix B). In the final validated questionnaire, the I-CVI of all items was 1, which is considered relevant (Yusoff, 2019). The S-CVI/UA and S-CVI/Ave were 0.72 and 0.91, which showed moderate and high validity, respectively (Polit & Beck, 2006).

**Table 2:** Content Validity Index Analysis

CVI	Result	Cut-off Criteria
I-CVI	Relevant: 24 items Eliminated: 9 items	1 (Yusoff, 2019)
S-CVI/UA	0.72	≥ 0.08 (Polit & Beck, 2006)
S-CVI/Ave	0.92	≥ 0.09 (Polit & Beck, 2006)

*Note.* I-CVI = Item-Content Validity Index; S-CVI/UA = Scale-Content Validity Index using the Universal Agreement method; S-CVI/Ave = Scale-Content Validity Index using the Average method

In the pilot study conducted, the average duration of completing the survey was 7 minutes and 15 seconds. Feedback from the six participants of the pilot testing mainly was “no changes to be done” in the form.

### PHASE II: QUESTIONNAIRE IMPLEMENTATION

#### PARTICIPANTS' DEMOGRAPHICS

A total of 45 eligible participants responded to the questionnaire, 75% of the targeted sample size. Of the 45 participants, 82% were female, 18% were male. Results also revealed that 82% belonged to the young adult group (18-35 years old) while 18% belonged to the middle adult group (36-55 years old). Most of the participants had less than three (33%) years of practice as a pediatric PT, followed by 6-10 years (29%); others had 3-5 (24%) years, 11-20 (9%) years, and more than 20 (4%) years of experience. Regarding telerehabilitation practice, 51% have been practicing for less than a year, while 49% have been practicing for 1-3 years. Findings further revealed that 51% were OMT users during telerehabilitation, while 49% were non-OMT users. All participants completed the survey without missing data. Table 3 illustrates the demographic characteristics of the participants.

**Table 3:** Demographic Characteristics of Survey Participants

Demographic Characteristics	Frequency (*n* = 45)	Percentage (%)
Gender		
Female	37	82%
Male	8	18%
Age		
18–35	37	82%
36–55	8	18%
> 55	0	0%
Years of practice as a pediatric PT		
< 3	15	33%
3–5	11	24%
6–10	13	29%
11–20	4	9%
> 20	2	4%
Years of practice as a pediatric PT using telerehabilitation		
< 1	23	51%
1–3	22	49%
>4	0	0%
Status of OMT Usage		
I use OMTs in telerehabilitation	23	51%
I do not use OMTs in telerehabilitation	22	49%

### COMMON OMTS IN PEDIATRIC TELEREHABILITATION

For the 23 (51%) participants who use OMTs, the survey asked them to list at least one to five OMTs they use during telerehabilitation. Findings revealed that the most common OMT used in pediatric telerehabilitation is GMFM (100%)— including both versions of GMFM-88 and GMFM-66, followed by PBS (30%). The third commonly used OMTs include the GAS, TGMD-2, Gross Motor Function Classification System (GMFCS), and self-made OMTs (13%). However, the researchers did not consider GMFCS an OMT and omitted the self-made OMTs.

Other OMTs used by 9% of the participants included the Alberta Infant Motor Scale (AIMS), Canadian Occupational Performance Measure (COPM), Infant/Toddler Sensory Profile (ITSP), Modified Timed Up and Go (MTUG), Test of Gross Motor Development (TGMD), Peabody Developmental Motor Scale first and second editions (PDMS-1, PDMS-2).

Moreover, other OMTs used by 4% of the participants included Affordances in the Home Environment for Motor Development (AHEMD), Brigance Inventory of Early Childhood, and Bruininks-Oseretsky Test of Motor Proficiency Second Edition (BOT-2), CPCHILD, Dyskinesia Rating Scale (DRS), Functional Independence Measure (FIM), Functional Status Scale (FSS), Observational Gait Scale (OGS), Pediatric Quality of Life Inventory (PedsQL), and Pediatric Reach Test (PRT).

Four percent also reported use of the School Functional Assessment (SFA), Sit to Stand Test (STS), Trunk Control Measurement Scale (TCMS), Timed Up and Down Stairs (TUDS), Timed Up and Go (TUG), and WeeFim.

Lastly, one participant (4%) listed the International Classification of Function Model (ICF Model) and gait analysis, which the researchers omitted. Percentages on the common OMTs do not equate to 100% since this was a multiple response item. Table 4 shows the frequency and percentages of the results.

**Table 4:** Common OMTs Used by Pediatric Telerehabilitation PTs

OMT	Frequency (*n* = 23)	Percentage (%)
Gross Motor Function Measure (GMFM)	23	100%
Pediatric Balance Scale (PBS)	7	30%
Goal Attainment Scale (GAS)	3	13%
Test of Gross Motor Development - Second Edition (TGMD-2)	3	13%
Gross Motor Function Classification System (GMFCS) ^a^	3	13%
Self-made OMTs ^b^	3	13%
Others ^c^	≤ 2	13%

a *Note*. Some participants listed GMFCS, but the researchers do not consider this as an OMT.

b Researchers omitted selfmade OMTs.

c Other OMTs cited by 2 PTs (9%) include AIMS, COPM, ITSP, MTUG, TGMD, PDMS-1, and PDMS-2; cited by 1 PT (4%) include AHMED, BOT-2, Brigance Inventory of Early Childhood, CPCHILD, DRS, FIM, FSS, ICF Model, OGS, PDMS-2, PedsQL, PRT, SFA, STS, TCMS, TUDS, TUG, WeeFim, and gait analysis.

### DETAILS OF OMT USAGE AND NON-USAGE

The OMT users also answered questions about details in their OMT usage. Of the 23 participants, 39% reported that OMT completion was required for all patients; it was routine for 35%, but not required. Thirteen percent said that it was only routine and not mandated for patients or clients who have certain types of conditions. Meanwhile, 9% of the participants reported sporadic completion, depending on different factors such as time, patient characteristics, etc.; and 4% required OMT use for patients with certain conditions.

In terms of the type of OMTs used, 96% of the participants reported using a combination of patient-reported outcome measures (PROMs) and observer-reported outcomes (ObsRO), while 4% only used ObsRO. For the format of OMTs, most of the OMT users (48%) used paper with PTs reviewing raw information from the paper version, while 39% utilized a computer when filling out a tool with summary scores reviewed by PTs. The remaining 13% used paper with analysis and scoring of information done through a scanner or computer data entry with the summary of scores reviewed by PTs.

Lastly, findings revealed that the top enablers for OMT use in the telerehabilitation setting were the criterion of being easily understood by patients, clients, and caregivers (87%), followed by the appropriateness of OMTs based on patient's type of conditions (83%). Other enablers agreed by 78% of the participants included ease of score interpretation by clinicians, usefulness for various purposes, and validity and reliability of OMTs. Seventy percent also reported quick completion of OMTs as an enabler. Percentages on the criteria for OMT selection do not equate to 100% since this was a multiple response item. Table 5 reports the full summary of the details in OMT use.

**Table 5:** Details in OMT Use

Statement	Frequency (*n* = 23)	Percentage (%)
POLICIES & PROCEDURES ON THE COMPLETION OF OMTS		
Mandated/required for all patients	9	39%
Routine for all patients/clients, but not mandated/required	8	35%
Routine, but not mandated, only for patients/clients who have certain types of conditions (e.g., cerebral palsy)	3	13%
Sporadic, depending on different factors such as time, patient's characteristics, etc.	2	9%
Mandated only for patients/clients who have certain types of conditions (e.g., cerebral palsy)	1	4%
POLICIES & PROCEDURES ON THE TYPE OF OMTS		
A combination of those that use patient/client self-report and observation of their performance	22	96%
Only those that use information derived from observation of patients'/clients' performance	1	4%
Only those that use information derived from patients'/clients' self-report.	0	0%
POLICIES & PROCEDURES ON OMT COMPLETION FORMAT		
Use paper, and therapists review the raw information from the paper version.	11	48%
Use the computer (no paper), and summary scores are reviewed by therapists.	9	39%
Using paper, analyzed/scored through scanner or computer data entry, and then summary scores are reviewed by therapists.	3	13%
Criteria used for OMT selection		
Easy for patients/clients/caregivers to understand.	20	87%
Most appropriate for the types of conditions seen in my practice setting.	19	83%
Easy for clinicians to understand/interpret meaning of scores and change in scores.	18	78%
Useful for a variety of purposes such as research, quality assurance, patient/client evaluation.	18	78%
Shown to be valid and reliable.	18	78%
Can be completed quickly.	14	61%

In contrast, 49% of the recruited participants reported that they do not use OMTs during telerehabilitation, with 55% still thinking about using it, 36% indicating a willingness to use it, and 9% reporting that they do not plan to use OMTs in the future.

Findings suggest that the main reason for the non-usage of OMTs during telerehabilitation is the difficulty for the stakeholders to complete OMTs independently (68%), followed by being confusing to stakeholders (45%), taking too much time for completion (36%), and making stakeholders anxious (36%).

Other reasons for non-usage of OMTs agreed to by 27% of the participants include being written in the English language and providing information that is too subjective. Moreover, 23% of the participants also reported that clinicians do not have the proper training for its usage and that OMTs require a high reading level for most stakeholders.

Eighteen percent of non-OMT users also reported that OMTs require more effort than they were worth. Additionally, 14% said that OMTs are difficult to interpret and take too much of a clinician's time to analyze, calculate, and score the information. Lastly, 9% reported that OMTs do not contain information that helps direct the treatment plan and are only helpful for research purposes. The reasons for the non-usage of OMTs do not summate to 100% since this was a multiple response item. Table 6 summarizes the reasons for the non-usage of OMTs in pediatric telerehabilitation

**Table 6:** Reasons for Non-usage of OMTs in Pediatric Telerehabilitation

Reasons	Frequency (*n* = 22)	Percentage (%)
Are difficult for patients/clients/caregivers to complete independently.	15	68%
Are confusing to patients/clients/caregivers.	10	45%
Take too much time for patients/clients/caregivers to complete.	8	36%
Make patients/clients/caregivers anxious.	8	36%
Are in English, a language in which many of my patients/clients/patients' caregivers are not fluent.	6	27%
Provide information that is too subjective to be useful.	6	27%
Require training that I do not have.	5	23%
Require too high a reading level for many patients/clients/caregivers.	5	23%
Require more effort than they are worth.	4	18%
Are difficult to interpret (e.g., do not know what the norms are, how score relates to severity, or what a clinically important change might be).	3	14%
Take too much of clinicians' time to analyze/calculate/score.	3	14%
Do not contain information that helps to direct the plan of care.	2	9%
Really only useful for research purposes.	2	9%

## DISCUSSION

### COMMON OMTS IN PEDIATRIC TELEREHABILITATION

Respondents reported that the most commonly used OMT by pediatric telerehabilitation PTs in the Philippines is the GMFM—including both versions of GMFM-88 and GMFM-66. Two studies support this finding as they reported GMFM as the most frequently used OMT from their surveys (Hanna et al., 2007; Knox et al., 2019). Similarly, Fay et al. (2018) cited GMFM as the second most used OMT in their study. Moreover, Thomason and Wilmarth (2015) mentioned GMFM as the most reported additional OMT used in school-based PT services in preschool, elementary, and middle-/high-school students. Russell et al. (2010) also conducted a before-after intervention study to assess the use of pediatric OMTs in clinical practice. At baseline, they found that most of the PTs were already familiar with GMFM, and after the 6-month intervention, all their recruited PTs were familiar with the tool.

The cited studies above occurred in an in-person setting. Currently, there is a lack of studies on the use of OMTs in pediatric telerehabilitation. However, Montecino (2021) suggested GMFM and PBS as feasible validated tools, consistent with our top two common OMTs, in a recent online rehabilitation medicine convention on the trends in pediatric physical therapy that highlighted various OMTs used in practice.

It was foreseeable that the GMFM was the most frequently listed tool due to its feasibility across various rehabilitation settings, including telerehabilitation (Alotaibi et al., 2014). In 2017, CanChild of McMaster University developed the GMFM App, containing the GMFM-88 and GMFM-66 versions, making it more accessible to use (McMaster University, 2021). Alotaibi et al. (2014) also reported that GMFM is practical and easy to administer without requiring expensive equipment. Moreover, Brunton and Bartlett (2011) stated that GMFM is the current “gold standard” to measure gross motor function in children with cerebral palsy (CP). Although it is a highly recommended supplemental OMT for CP, researchers reported that GMFM is a feasible generic tool for different pediatric conditions. Studies recommend using GMFM for children with Down syndrome, traumatic brain injury (TBI), spinal muscular atrophy (SMA), myotonic dystrophy, and mitochondrial disease (National Institute of Neurological Disorders and Stroke, 2019; Russell et al., 2013).

Moreover, GMFM's good psychometric properties may also contribute to its widespread use by PTs. Adair et al. (2012) reported that both versions of GMFM have excellent levels of reliability and internal consistency in children with CP, SMA, and Down syndrome. Several articles similarly support the construct, criterion-related, and content validity of the same population. Findings also suggest that both versions of GMFM are responsive in terms of minimum clinically important difference (MCID), effect size, changes after interventions, and receiver operating curves (i.e., clinical specificity and sensitivity) in children with CP, Down syndrome, and TBI (Adair et al., 2012).

While previous literature has not cited PBS as a frequently used OMT, studies have reported it as a reliable measure of functional balance in school-age children, CP, spina bifida, and the pediatric population in general (Boyd, 2015; Franjoine et al., 2003). Most of the respondents may have been using PBS since it is easy to administer and score—a therapist may complete the administration and scoring of PBS in less than 20 min. Additionally, PBS does not require any special training and the use of specialized and expensive equipment (Boyd, 2015; Franjoine et al., 2003). PBS also shows an excellent test-retest and interrater/intrarater reliability for pediatric disorders. Researchers similarly reported cut-off scores, normative data, and floor/ceiling effects of PBS (Boyd, 2015).

The participants reported that the third commonly used OMTs are GAS and TGMD-2. These findings are consistent since Knox et al. (2019) and Thomason and Wilmarth (2015) similarly cited GAS and TGMD-2 as frequently reported tools, respectively. Both OMTs likewise show good psychometric properties for pediatric disorders (Pham et al., 2020; Rey et al., 2020). Additionally, a possible explanation for GAS being a common OMT in telerehabilitation is its availability in an app entitled GOALed, which has a streamlined approach and is easier to use (Gaffney et al., 2019).

Finally, Towns and Rosenbaum (2018) stated that GMFCS is a classification system for children with CP that describes their gross motor functioning levels and not an OMT. This conflicting evidence prompted the researchers to disregard GMFCS as an OMT. Furthermore, some participants reported using self-made OMTs, which the researchers omitted since they are not validated and have no proven psychometric properties to ensure the credibility and reliability of a patient's status and improvement. Additionally, the group disregarded the ICF Model as an OMT since Steiner et al. (2002) classified this as a conceptual framework for categorizing and comprehending a patient's disease. The researchers similarly omitted gait analysis as Rabatin et al. (2020) considered it an examination procedure rather than an OMT.

### DETAILS OF OMT USAGE AND NON-USAGE

When using OMTs, the top enabler agreed by this study's participants is ease for patients/clients/caregivers to understand. This finding is somehow inconsistent with the data reported by Hanna et al. (2007). They stated that finding measures that meet clients' needs had the most substantial influence on standardized clinical measures. Nevertheless, the participants of this study also considered OMT applicability based on a patient's conditions as the third enabler of OMT usage. Furthermore, the other enablers agreed to by this study's participants, (i.e., validity and reliability of OMTs, and quick completion of OMTs), coincide with the study's findings conducted by Fay et al. (2018). They reported that most respondents consider the ease of completion and the assessment tools' quality/statistical/psychometric properties. Likewise, these mentioned references report the factors influencing the selection of assessment tools in in-person services because of the current lack of studies on the facilitators and barriers of OMT use in telerehabilitation.

Conversely, most of the PTs agreed that difficulty for the stakeholders to complete OMTs independently and confusion to stakeholders are the main reasons for the non-usage of OMTs in telerehabilitation. These are consistent with the study by King et al. (2011). They reported that PTs might feel that OMTs are not appropriate to clients' circumstances or goals or are too confusing or difficult for clients to complete leading to lack of usage. Furthermore, Aberdeen et al. (2019) noted that the main barriers to OMT use are difficulty remembering to administer and uncertainty about which PROMs to use, which this study's participants similarly agreed to.

Two studies also support taking too much time for completion as one of the primary reasons for the lack of OMT usage (Aberdeen et al., 2019; Fay et al., 2018). Fay et al. (2018) said that the assessment/time limitation length was a problem for more than half of their respondents. This reason was further supported by King et al. (2011), stating that therapist time constraints is the most commonly cited reason for the lack of standardized outcome measures. The other less influential factors noted in this study, namely clinicians not having the proper training for its usage and difficulty interpreting results, were also found to be less influential factors in the study of Fay et al. (2018).

## LIMITATIONS

The primary limitation noted in this quantitative study was the lack of participants recruited during Phase II. The limited number of Filipino pediatric PTs who practice telerehabilitation in the Philippines caused difficulty in recruiting participants. Consequently, the unavailability of potential respondents to participate in this study yielded a deficit in meeting the calculated sample size of 60. Hence, generalization of the acquired results to the population of this quantitative study may not be applicable.

## IMPLICATIONS AND RECOMMENDATIONS

This study enumerated commonly used OMTs in pediatric telerehabilitation and described their characteristics that make it easy for PTs to use due to their applicability in the online setting. PTs offering telerehabilitation to the pediatric population may want to consider using the listed common OMTs in this study to make their examination more objective.

These findings support the need for further research to establish a clinical guideline in pediatric telerehabilitation in the Philippines. Researchers recommend conducting a qualitative study to elaborate the facilitators, barriers, attitudes, and perceptions on the usage and non-usage of OMTs in telerehabilitation and explain the experiences of Pediatric PTs. Further exploration of the frequently used OMTs, including PTs outside the Philippines with increased sample size, may also be conducted for more generalized results.

## CONCLUSION

The commonly used OMTs in pediatric telerehabilitation are the GMFM, PBS, GAS, and TGMD-2. Survey reports from PTs indicate that the main reason for OMT use is the ease of understanding by the stakeholders. However, the non-users agreed that OMTs are difficult for stakeholders to complete independently. In terms of generalizability, the low number of respondents limits the results. Thus, further research would help promote evidence-based PT practice and enhance the quality of patient care for pediatric telerehabilitation in the Philippines.

## APPENDIX A FINAL VALIDATED QUESTIONNAIRE

Standardized Outcome Measures in Physical Therapist Practice

*Survey adapted from Dr. Jette et al. 2009*

**INSTRUCTIONS:** This adapted survey has 15 questions divided into 5 sections for OMT users & 8 questions divided into 3 sections for Non-OMT users. All questions are required to be answered in the context of **TELEREHABILITATION ONLY.** You must complete this survey in one-sitting and you may only submit it once.

**Section 1: Demographics**

Please provide some information about yourself by filling out or checking the relevant box under each item.

1. Email address:
2. Age:
3. Your sex
  □ Female   □ Male
4. The number of years you have practiced as a pediatric physical therapist
  □ < 3.   □ 3-5   □ 6-10   □ 11-20   □ >20
5. Years of experience as a pediatric physical therapist using **TELEREHABILITATION**
  □ < 1   □ 1-3   □ 4-6   □ 7-10   □ >10

**Section 2: Usage or non-usage of OMTs**

6. Status of OMT Usage
  □ I use OMTs in telerehab
  □ I do not use OMTs in telerehab
  □ I only use OMTs in an in-clinic setting, not in telerehab
  □ I use OMTs both in an in-clinic setting and telerehab

**For OMT Users**

**Section 3: Policies & Procedures for OMT use in Practice Setting**

The following section asks about policies or procedures used in **TELEREHAB** for collecting information from OMTs. Please check only one box for each item.

7. In my practice setting, completion of health status questionnaires is
  □ Mandated/required for all patients.
  □ Mandated only for patients/clients who have certain types of conditions (e.g., cerebral palsy).
  □ Routine for all patients/clients, but not mandated/required.
  □ Routine, but not mandated, only for patients/clients who have certain types of conditions (e.g., cerebral palsy).
  □ Sporadic, depending on different factors such as time, patient's characteristics, etc.
8. In my practice setting, the types of health status questionnaires used include
  □ Only those that use information derived from patients'/clients' self-report.
  □ Only those that use information derived from observation of patients'/clients' performance.
  □ A combination of those that use patient/client self-report and observation of their performance.
9. In my practice setting, health status questionnaires are completed
  □ Using paper, and therapists review the raw information from the paper version.
  □ Using paper, analyzed/scored through scanner or computer data entry, and then summary scores are reviewed by therapists.
  □ Using the computer (no paper), and summary scores are reviewed by therapists.

**Section 4: List of OMTs used in Telerehab**

Please list down the OMTs you use during **TELEREHAB ONLY.** Indicate which, if any, are your own “home-grown/self-made” questionnaires. Kindly spell out the name for each and place N/A for unused blanks. **(Note:** Currently, there are no developed pediatric OMTs specifically for telerehab, you may list down OMTs you previously used in an in-clinic setting and are now using for telerehab.)

(E.g., Gross Motor Function Measure-88, Pediatric Evaluation of Disability and Inventory, Edinburgh Visual Gait Analysis)

10. Kindly list one (1) OMT:________
11. List an OMT or type N/A:________
12. List an OMT or type N/A:________
13. List an OMT or type N/A:________
14. List an OMT or type N/A:________

**Section 5: Criteria Used for OMT selection**

15. Please indicate the criteria used for selecting the OMTs you use in **TELEREHAB ONLY.** Please check all that apply.
  □ Can be completed quickly.
  □ Easy for patients/clients/caregivers to understand.
  □ Easy for clinicians to understand/interpret meaning of scores and change in scores.
  □ Shown to be valid and reliable.
  □ Seem to be the most common ones used in physical therapist practice.
  □ Useful for a variety of purposes such as research, quality assurance, patient/client evaluation.
  □ Most appropriate for the types of conditions seen in my practice setting.

**For Non-OMT users**

**Section 3: Reasons for non-usage of OMTs**

This section of the survey contains items that ask for the reason you do not use OMTs during telerehab. Please check all that apply.

7. I do not use OMTs during telerehabilitation because they:
  □ Are confusing to patients/clients/caregivers.
  □ Are difficult for patients/clients/caregivers to complete independently.
  □ Require too high a reading level for many patients/clients/caregivers.
  □ Are in English, a language in which many of my patients/clients/pt's caregivers are not fluent.
  □ Make patients/clients/caregivers anxious.
  □ Take too much time for patients/clients/caregivers to complete.
  □ Take too much of clinicians' time to analyze/calculate/score.
  □ Provide information that is too subjective to be useful.
  □ Require more effort than they are worth.
  □ Do not contain information that helps to direct the plan of care.
  □ Are difficult to interpret (e.g., do not know what norms are, how score relates to severity, or what a clinically important change might be).
  □ Require training that I do not have.
  □ Are really only useful for research purposes.

8. I am planning to use OMTs during telerehab in the near future
  □ Yes □ No □ Maybe

## APPENDIX B I-CVI CALCULATIONS

**Table:**

Item	Description	CVI	Interpretation
Item 10	Kindly list one (1) OMT	1	Relevant
Item 11	List an OMT or type N/A	1	Relevant
Item 12	List an OMT or type N/A	1	Relevant
Item 13	List an OMT or type N/A	1	Relevant
Item 14	List an OMT or type N/A	1	Relevant
Statement 1	Please indicate the criteria used for selecting the OMTs you use in TELEREHAB ONLY. Please check all that apply: [Can be completed quickly ]	1	Relevant
Statement 2	Please indicate the criteria used for selecting the OMTs you use in TELEREHAB ONLY. Please check all that apply: [Easy for patients/clients/caregivers to understand]	1	Relevant
Statement 3	Please indicate the criteria used for selecting the OMTs you use in TELEREHAB ONLY. Please check all that apply: [Easy for clinicians to understand/interpret the meaning of scores and change in scores]	1	Relevant
Statement 4	Please indicate the criteria used for selecting the OMTs you use in TELEREHAB ONLY. Please check all that apply: [Shown to be valid and reliable]	1	Relevant
Statement 5	Please indicate the criteria used for selecting the OMTs you use in TELEREHAB ONLY. Please check all that apply: [Seem to be the most common ones used in physical therapist practice]	0.67	Eliminated
Statement 6	Please indicate the criteria used for selecting the OMTs you use in TELEREHAB ONLY. Please check all that apply: [Useful for a variety of purposes such as research, quality assurance, patient/client evaluation]	1	Relevant
Statement 7	Please indicate the criteria used for selecting the OMTs you use in TELEREHAB ONLY. Please check all that apply: [Can be analyzed electronically (scanner, computer, etc.)]	0.67	Eliminated
Statement 8	Please indicate the criteria used for selecting the OMTs you use in TELEREHAB ONLY. Please check all that apply: [Most appropriate for the types of conditions seen in my practice setting]	1	Relevant
Statement 1	I do not use OMTs during telerehabilitation because they [Are confusing to patients/clients/caregivers]	1	Relevant
Statement 2	I do not use OMTs during telerehabilitation because they [Are difficult for patients/clients/caregivers to complete independently]	1	Relevant
Statement 3	I do not use OMTs during telerehabilitation because they [Require too high a reading level for many patients/clients/caregivers]	1	Relevant
Statement 4	I do not use OMTs during telerehabilitation because they [Are in English, a language in which many of my patients/clients/pt's caregivers are not fluent]	1	Relevant
Statement 5	I do not use OMTs during telerehabilitation because they [Are not sensitive to the cultural/ethnic concerns of many patients/clients/caregivers]	0.67	Eliminated
Statement 6	I do not use OMTs during telerehabilitation because they [Make patients/clients/caregivers anxious]	1	Relevant
Statement 7	I do not use OMTs during telerehabilitation because they [Take too much time for patients/clients/caregivers to complete]	1	Relevant
Statement 8	I do not use OMTs during telerehabilitation because they [Take too much of clinicians' time to analyze/calculate/score]	1	Relevant
Statement 9	I do not use OMTs during telerehabilitation because they [Provide information that is too subjective to be useful]	1	Relevant
Statement 10	I do not use OMTs during telerehabilitation because they [Require more effort than they are worth]	1	Relevant
Statement 11	I do not use OMTs during telerehabilitation because they [Do not contain information that helps to direct the plan of care]	1	Relevant
Statement 12	I do not use OMTs during telerehabilitation because they [Are difficult to interpret (e.g., do not know what norms are, how score relates to severity, or what a clinically important change might be)]	1	Relevant
Statement 13	I do not use OMTs during telerehabilitation because they [Do not contain the types of items or questions that are relevant for the types of patients/clients I see]	0.67	Eliminated
Statement 14	I do not use OMTs during telerehabilitation because they [Often do not get completed at discharge, so are not useful for determining patients'/clients' response to treatment]	0.67	Eliminated
Statement 15	I do not use OMTs during telerehabilitation because they [Require training that I do not have]	1	Relevant
Statement 16	I do not use OMTs during telerehabilitation because they [Cost too much]	0.67	Eliminated
Statement 17	I do not use OMTs during telerehabilitation because they [Require a support structure that I do not have (e.g., internet connection, electronic devices, staffing)]	0.67	Eliminated
Statement 18	I do not use OMTs during telerehabilitation because they [Are really only useful for research purposes]	1	Relevant
Statement 19	I do not use OMTs during telerehabilitation because they [Are not relevant because my practice involves consultation, case management, or discharge planning only]	0.67	Eliminated
Statement 20	I do not use OMTs during telerehabilitation because they [Other: ______________]	0.67	Eliminated
